# Safety and Effectiveness of Ninjin'yoeito: A Utilization Study in Elderly Patients

**DOI:** 10.3389/fnut.2019.00014

**Published:** 2019-02-19

**Authors:** Shinichi Suzuki, Fumitaka Aihara, Miho Shibahara, Katsutaka Sakai

**Affiliations:** ^1^Pharmaceutical Division, Kracie Pharma Ltd., Tokyo, Japan; ^2^Safety Management Department, Kracie Pharma Ltd., Tokyo, Japan; ^3^Kampo Research Laboratory, Kracie Pharma Ltd., Takaoka, Japan

**Keywords:** ninjin'yoeito, post-marketing surveillance studies, elderly patient, basic checklist, frailty

## Abstract

Post-marketing surveillance studies of traditional Japanese medicine in Japan are limited, and currently there are no data for Ninjin'yoeito, which is often used for the elderly because of its efficacy. In this study, we aimed to investigate the post-marketing safety and efficacy of Ninjin'yoeito in elderly patients over 65 years of age in clinical practice in Japan. This survey was an open-label, non-comparative, prospective, multicenter, post-marketing survey conducted at 383 centers between February 2016 and March 2017. In the safety analysis of 808 patients, adverse reactions were reported in 25 patients (3.1%), most of whom had gastrointestinal disorders (2.1%). In the efficacy analysis, Ninjin'yoeito was found to significantly improve visual analog scale scores in fatigue/malaise and anorexia at weeks 8, 16, and 24, and weeks 8 and 24 after commencement of treatment, respectively. In addition, the Basic Checklist created by the Ministry of Health, Labor and Welfare of Japan was used as a secondary survey item. The proportion of patients expected to require nursing care significantly decreased after 24 weeks compared with the baseline in four domains (activities of daily living, motor function, oral function, and depression). On the basis of physician assessment, Ninjin'yoeito was rated as “effective” or “moderately effective” in 486 (90.5%) of 537 cases. As the checklist contains many aspects of frailty, Ninjin'yoeito might be beneficial in preventing frailty. The findings of the present study indicate the safety of Ninjin'yoeito in aged patients, although further integrated clinical trials are necessary to examine its efficacy.

## Introduction

Aging is the balance between physiological damage and repair, and the ability to repair physiological damage is assumed to decline with age. The accumulation of physiological damage is presumed to increase the risk of frailty, such as loss of muscle volume, depression, and anorexia in elderly people. In particular, those individuals who have physiological and psychological complications (such as cardiovascular disease, diabetes, hypertension, cancer, and cognitive impairment) are reported to have a high risk of developing frailty-related symptoms.

Ninjin'yoeito (NYT, Ren-Shen-Yang-Rong-Tang in Chinese medicine) is a traditional Japanese medicine (Kampo medicine) that is used for individuals with deteriorated physical or psychiatric conditions, particularly among the elderly. A recent clinical study revealed that NYT has the potential to improve cognitive outcome and Alzheimer's disease-related depression in Alzheimer's patients (1). In addition, NYT is also reported to improve anorexia and loss of grip strength in elderly individuals (2, 3). Furthermore, NYT is reported to improve cancer cachexia in patients with multiple myeloma and fatigue in cancer survivors (4, 5). On the basis of these reports, we hypothesized that NYT would be the candidate drug for the treatment of the frailty symptoms observed in elderly individuals. However, to date no post-marketing survey or other relevant studies have been performed to determine the incidence of adverse drug reactions (ADRs) or the efficacy of NYT. Thus, in the present study, we conducted a post-marketing survey in elderly patients to evaluate the safety and effectiveness of NYT.

## Materials and Methods

### Study Design and Subjects

This was an open-label, non-comparative, prospective, multicenter, post-marketing survey to investigate the safety, and efficacy of NYT for self-ambulatory outpatients aged 65 years or older with at least one of the following indications: deterioration in constitution after disease, fatigue/malaise, anorexia, night sweat, coldness of limbs, and anemia. Subjects visited hospitals or clinics at baseline (week 0), and thereafter at weeks 8, 16, and 24. Written informed consent to participate in the study was obtained from each subject or his or her legally authorized representative. The data were collected from February 2016 to March 2017.

### Plant Materials and Preparation of the Extract

NYT, produced by Kracie Pharma Ltd, is composed of the following 12 dried medicinal herbs: Rehmannia root, Japanese Angelica root, Atractylodes rhizome, Poria sclerotium, Ginseng, Cinnamon bark, Peony root, Citrus Unshiu peel, Polygala root, Astragalus root, Schisandra fruit, and Glycyrrhiza (Table 1). Each plant material was identified based on external morphology and authenticated by marker compounds of plant specimens according to the method of the Japanese Pharmacopeia and company's standards.

**Table 1:** Medicinal herb composition of Ninjin'yoeito.

**Common name**	**Weight (g)**
Rehmannia root	4
Japanese angelica root	4
Atractylodes rhizome	4
Poria sclerotium	4
Ginseng	3
Cinnamon bark	2.5
Peony root	2
Citrus unshiu peel	2
Polygala root	2
Astragalus root	1.5
Schisandra fruit	1
Glycyrrhiza	1

*7.5 g (clinical daily dose) of this herbal preparation contains 6,700 mg of dried extract obtained from a mixture of above-mentioned crude drugs*.

### Survey Item

Patient characteristics [age, sex, height, body weight, body mass index (BMI), comorbidities, and concomitant medications] and the usage of NYT (indications for use, administration) were evaluated.

### Safety

The occurrence of adverse events (including abnormal laboratory values) during the use of NYT, were evaluated by recording the events, date of onset, severity, outcome (including date of outcome), measures taken, subsequent use of NYT, and a causal relationship with NYT. In addition, adverse events for which drug causality could not be excluded are considered as ADRs in this study. The ADRs were classified on the basis of system organ classes (SOC) and preferred terms (PT) of MedDRA/J ver. 20.0. The incidence of ADRs was calculated by the equation as follows:

$$\begin{array}{l} \text{The incidence }\left(\text{\%}\right)=\text{number of patients with SDRs}/\text{safety}\\ \text{ population }\times \;100 \end{array}$$

### Efficacy

At baseline and each of the subsequent 8-week-interval follow-up visits, the following parameters were assessed: (1) fatigue/malaise and anorexia, using a visual analog scale (VAS), (2) Basic Checklist, (3) severity of the decline in constitution after disease, night sweat, coldness of limbs, and anemia, which were rated by treating physicians on a 4-point scale (3, severe; 2, moderate; 1, mild; and 0, none), (4) body weight, (5) BMI, (6) overall improvement, which was evaluated at the end of the survey by treating physicians on a 4-point scale (3, effective; 2, moderately effective; 1, ineffective; and 0, unevaluable) based on their comprehensive assessment of signs and symptoms noted during the follow-up period.

The Basic Checklist is a questionnaire comprising 25 items (Table 2), which was created by the Ministry of Health, Labor, and Welfare of Japan. It represents a unique screening test used to assess and predict the risk of frailty. According to the criteria, a person with either a total score of ≥10 for questions 1–20 (activities of daily living), ≥3 for questions 6–10 (motor function), = 2 for questions 11 and 12 (nutrition), ≥2 for questions 13–15 (oral function), or ≥2 for questions 21–25 (depression) was considered to be in need of care dependency.

**Table 2:** Basic checklist.

**No**	**Questions**	**Answer**	
1	I usually take the bus or train when going out.	□0. YES	□1. NO
2	I usually buy daily necessities myself.	□0. YES	□1. NO
3	I usually withdraw and deposit money myself.	□0. YES	□1. NO
4	I sometimes visit my friends.	□0. YES	□1. NO
5	I sometimes turn to my family or friends for advice.	□0. YES	□1. NO
6	I usually climb stairs without using any handrails or wall for support.	□0. YES	□1. NO
7	I usually stand up from a chair without any aids.	□ 0. YES	□1. NO
8	I usually walk for about 15 min without stopping.	□0. YES	□1. NO
9	I fell in the past year.	□1. YES	□0. NO
10	I am seriously concerned about falling.	□1. YES	□0. NO
11	I have lost 2 kg or more in the past 6 months.	□1. YES	□0. NO
12	Height: cm, Weight: kg, BMI: kg/m^2^. If BMI is <18.5, this item is scored.	□1. YES	□0. NO
13	It is more difficult to eat solid food now compared to 6 months ago.	□1. YES	□0. NO
14	I sometimes choke when drinking something, such as tea or soup.	□1. YES	□0. NO
15	I am often concerned about my dry mouth.	□1. YES	□0. NO
16	I go out at least once a week.	□0. YES	□1. NO
17	I go out less frequently compared to last year.	□1. YES	□0. NO
18	My family or friends point out my memory loss. e.g., “You always ask the same question over and over again.”	□1. YES	□0. NO
19	I make a call by looking up phone number.	□0. YES	□1. NO
20	I sometimes lose track of the date.	□1. YES	□0. NO
21	In the last 2 weeks, I have felt lack of fulfillment in my life.	□1. YES	□0. NO
22	In the last 2 weeks, I have felt a lack of joy when doing the things I used to enjoy.	□1. YES	□0. NO
23	In the last 2 weeks, I have felt difficulty in doing what I could do easily before.	□1. YES	□0. NO
24	In the last 2 weeks, I have felt helpless.	□1. YES	□0. NO
25	In the last 2 weeks, I have felt tired without a reason.	□1. YES	□0. NO

### Statistical Analysis

Quantitative data, including age and height, are shown as mean ± standard deviation (SD) values. The categorical data, including sex, comorbidities, concomitant medications, and drug usage are shown in terms of frequency. VAS and severity scores for each disease were analyzed using a paired *t*-test with Bonferroni adjustment. Body weight and BMI were analyzed using a paired *t*-test. The scores in the five domains of the Basic Checklist (activities of daily living, motor function, nutrition, oral function, and depression) were evaluated using the McNemar test. The statistical software “EZR” (Easy R), which is based on R and R commander (6), was used for statistical analysis. A value of *p* < 0.05 was considered statistically significant.

### Information on Medical Ethics

This survey was conducted according to the Ordinance on Standards for Conducting Post-marketing Surveillance and Studies on Drugs (Japanese Ministry of Health, Labor and Welfare Ordinance No. 171, issued on December 20, 2004).

## Results

### Patient Disposition

**Figure 1:**
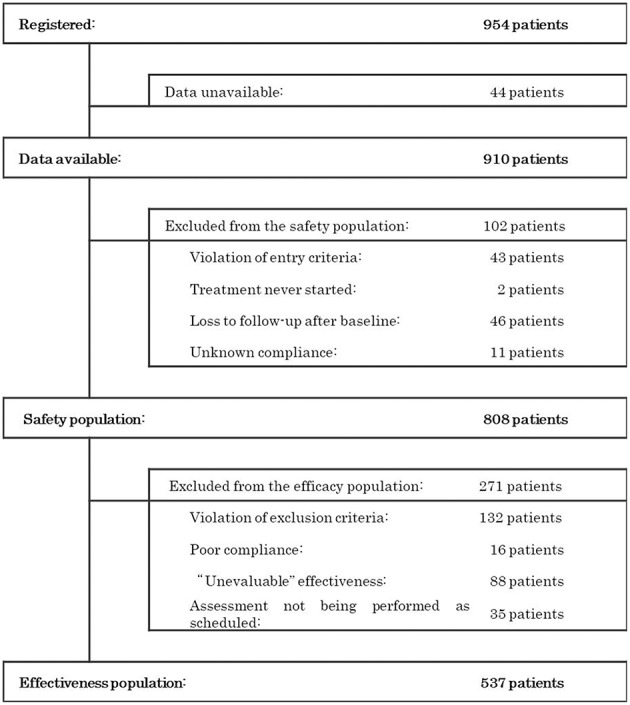
Patient disposition.

During the survey period, 954 patients were registered at 383 contracted medical facilities nationwide. From these patients, we collected 910 survey forms. The disposition of the patients is shown in Figure 1. Of the 910 patients surveyed, 808 were included in the population evaluated for safety after excluding 102 patients due to the following factors: violations of entry criteria (*n* = 43), treatment never started (*n* = 2), loss to follow-up after baseline (*n* = 46), and unknown compliance (*n* = 11). Among those individuals in the safety population, 271 patients were excluded due to violations of the exclusion criteria (*n* = 132), poor compliance (*n* = 16), an overall improvement rating of “unevaluable” (*n* = 88), and assessment not being performed as scheduled (*n* = 35), leaving 537 patients in the population used to assess effectiveness.

### Patient Characteristics

The safety analysis assessed 210 males (frequency, 26.0%) and 598 females (74.0%) aged 77.8 ± 7.4 years (mean ± SD), with an average height of 153.5 ± 8.9 cm. Of the 808 patients, 538 patients (66.6%) had comorbidities and 262 patients (32.4%) did not, and 664 patients (82.2%) received concomitant medications and 130 patients (16.1%) did not (Table 3).

**Table 3:** Patient characteristics.

		**Total**	**Male**	**Female**
Safety population		808	210 (26.0%)	598 (74.0%)
Age (years)	Mean ± SD	77.8 ± 7.4	77.5 ± 8.9	78.0 ± 7.4
	Median	78	78	78
	Range	65 ~ 97	65 ~ 96	65 ~ 97
Height (cm)	Mean ± SD	153.5 ± 8.9 (*n* = 767)	163.7 ± 6.9 (*n* = 200)	150.0 ± 6.3 (*n* = 567)
	Median	153.0	164.0	150.0
	Range	129 ~ 180	130 ~ 180	129 ~ 166
Body weight (kg)	Mean ± SD	50.3 ± 9.9 (*n* = 743)	58.1 ± 11.2 (*n* = 196)	47.5 ± 8.0 (*n* = 547)
	Median	49.0	58.9	42.0
	Range	25 ~ 88	34.9 ~ 88	25 ~ 82
BMI (kg/m^2^)	Mean ± SD	21.2 ± 3.2 (*n* = 738)	21.6 ± 3.2 (*n* = 195)	21.2 ± 3.1 (*n* = 543)
	Median	21.4	22.1	21.1
	Range	12.4 ~ 34.0	14.7 ~ 31	12.4 ~ 34.0
Comorbidities	Available	538 (66.6%)	140 (66.7%)	398 (66.6%)
	Not available	262 (32.4%)	69 (32.9%)	193 (32.3%)
	Unknown	8 (1.0%)	1 (0.4%)	7 (1.1%)
Concomitant medications	Available	664 (82.2%)	161 (76.7%)	503 (84.1%)
	Not available	130 (16.1%)	45 (21.4%)	85 (14.2%)
	Unknown	14 (1.7%)	4 (1.9%)	10 (1.7%)

The reasons for the use of NYT were fatigue/malaise in 589 cases (41.1%), coldness of limbs in 271 cases (18.9%), anorexia in 253 cases (17.6%), deteriorated constitution after disease in 189 cases (13.2%), anemia in 72 cases (5.0%), and night sweat in 60 cases (4.2%). A total of 731 patients (90.5%) received the maximum daily dose of 7.5 g, and 616 (76.2%) and 163 (20.2%) patients followed a twice daily and three times-daily dosing schedule, respectively (Table 4).

**Table 4:** Drug usage.

		***n* (%)**
Indications for use	Deteriorated constitution after disease	189 (13.2%)
	Fatigue/malaise	589 (41.1%)
	Anorexia	253 (17.6%)
	Night sweats	60 (4.2%)
	Coldness of limbs	271 (18.9%)
	Anemia	72 (5.0%)
Dosage/day	2.5 g	12 (1.5%)
	3.75 g	17 (2.1%)
	4 g	1 (0.1%)
	5 g	46 (5.7%)
	7.5 g	731 (90.5%)
	12 g	1 (0.1%)
Administration	Once-daily	29 (3.6%)
	Twice-daily	616 (76.2%)
	Three times-daily	163 (20.2%)

*Indications for use had multiple answers. The total number of responses was 1,434. ():frequency*.

### Safety

The incidence of ADRs among the surveyed patients is presented in Table 5A. In the safety analysis (*n* = 808), 31 incidents of ADRs occurred in 25 patients, with an incidence of 3.1%. Among these 25 patients, five patients had more than two types of ADRs. The most common ADRs were gastrointestinal disorders occurred in 17 patients (2.10%), including five cases of nausea, four of abdominal discomfort, three of diarrhea, and two of constipation, and so on. Besides them, three each of metabolism and nutritional disorders, skin and subcutaneous tissue disorders, and general disorders and administration site conditions, together with one each of psychiatric disorders, vascular disorders, and hepatobiliary disorders were recorded in this study (Table 5B).

**Table 5:** Incidence of adverse drug reactions (ADRs)^*^.

	***n* or %**
Safety population (1)	808
Patients with ADRs (2)	25
ADR cases	31
ADR incidence [(2)/(1) × 100]	3.1%

* *Including the adverse events for which drug causality could not be excluded*.

**Table 5B:** Incidence of individual type of adverse drug reactions (ADRs).

**SOC**	***n* (%)**	**PT**	***N* (%)**
Metabolism and nutrition disorders	3 (0.37%)	Decreased appetite	2 (0.25%)
		Hypokalaemia	1 (0.12%)
Psychiatric disorders	1 (0.12%)	Anxiety	1 (0.12%)
Vascular disorders	1 (0.12%)	Hypertension	1 (0.12%)
Gastrointestinal disorders	17 (2.10%)^*^	Nausea	5 (0.62%)
		Abdominal discomfort	4 (0.50%)
		Diarrhea	3 (0.37%)
		Constipation	2 (0.25%)
		Eructation	1 (0.12%)
		Abdominal pain upper	1 (0.12%)
		Feces discolored	1 (0.12%)
		Vomiting	1 (0.12%)
		Others	1 (0.12%)
Hepatobiliary disorders	1 (0.12%)	Cholecystitis^**^	1 (0.12%)
Skin and subcutaneous tissue disorders	3 (0.37%)	Pruritus	1 (0.12%)
		Eczema	1 (0.12%)
		Rash	1 (0.12%)
General disorders and administration site conditions	3 (0.37%)	Chest discomfort	1 (0.12%)
		Malaise	1 (0.12%)
		Oedema	1 (0.12%)

The ADRs were classified on the basis of system organ class (SOC) and preferred terms (PT) of MedDRA/J ver. 20.0. n, number of patients; N, number of cases; (), the incidence was calculated by the equation, the rate (%) = number of patients/safety population × 100.

* Among 17 patients, two patients have two types of ADRs.

** *Causation was not established*.

The time period before event onset was as follows; ≤1 week in 10 cases; ≤2 weeks in 12 cases; ≤1 month in 17 cases; and ≤2 months in 21 cases (cumulative numbers). The result indicated that approximately 70% of the reported ADRs occurred within the first 2 months of treatment. In terms of severity, there was one case of cholecystitis leading to hospitalization (with an unknown causal relationship with NYT) and one case of non-mild constipation (with a causal relationship with NYT); the remaining 29 cases were classified as mild. The outcome was reported as “recovered” in 20 cases and “recovering” in eight cases.

### Efficacy

#### Changes in Symptom Scores on the VAS and Severity Scale

**Figure 2:**
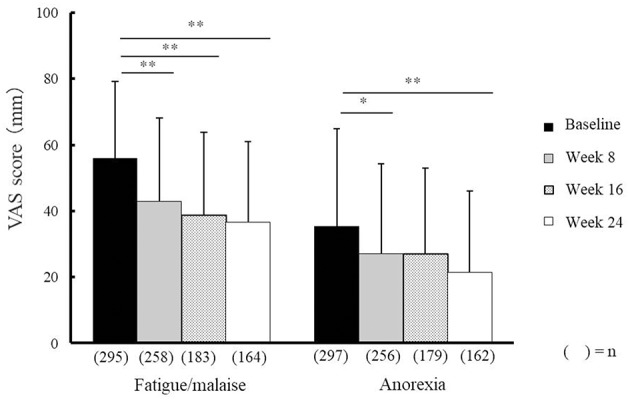
Effectiveness of Ninjin'yoeito against fatigue/malaise and anorexia. Changes in VAS scores for fatigue/malaise and anorexia after Ninjin'yoeito (NYT) administration, Data are presented as the mean ± standard deviation, ^*^p < 0.05 and ^**^p < 0.01 compared with the baseline as determined using the Bonferroni method. VAS, visual analog scale; (*n*), number of patients.

**Figure 3:**
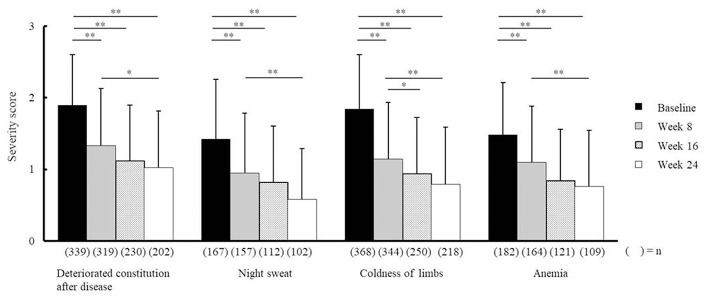
Effectiveness of Ninjin'yoeito according to physician assessment. Changes in severity scores for deteriorated constitution after disease, night sweat, coldness of limbs, and anemia after Ninjin'yoeito (NYT) administration, Data are presented as the mean ± standard deviation, ^*^p < 0.05 and ^**^p < 0.01 compared with the baseline as determined using the Bonferroni Method. (*n*), number of patients.

Compared with baseline scores, NYT significantly improved VAS scores relating to fatigue/malaise and anorexia at weeks 8, 16, and 24, and weeks 8 and 24, respectively (Figure 2). In addition, severity scores for deteriorated constitution after disease, night sweat, coldness of limbs, and anemia significantly improved at weeks 8, 16, and 24 compared with those at baseline (Figure 3). Furthermore, the severity scores for these symptoms were significantly improved at week 24 compared with those at week 8, and the score for coldness of limbs was significantly improved at week 16 compared with that at week 8.

#### Change in Body Weight and BMI

Body weight and BMI were analyzed in 275 patients whose data at both baseline and week 24 were determined. Mean body weight and BMI increased significantly from 49.4 ± 9.0 kg and 20.9 ± 2.6 kg/m^2^ at baseline to 50.0 ± 9.0 kg and 21.2 ± 2.6 kg/m^2^ at week 24, respectively (*n* = 275, *p* < 0.01 for both). The mean body weight and BMI of males was 58.1 ± 9.0 kg and 21.4 ± 2.7 kg/m^2^ at baseline and 58.4 ± 9.5 kg and 21.5 ± 2.8 kg/m^2^ at 24 weeks, respectively (*n* = 68: no significant difference for both).The mean body weight and BMI of females was 46.5 ± 6.9 kg and 20.7 ± 2.5 kg/m^2^ at baseline and 47.2 ± 6.8 kg and 21.0 ± 2.5 kg/m^2^ at 24 weeks, respectively (*n* = 207, *p* < 0.01 for both).

#### Change in the Scores Assessed Using the Basic Checklist

**Figure 4:**
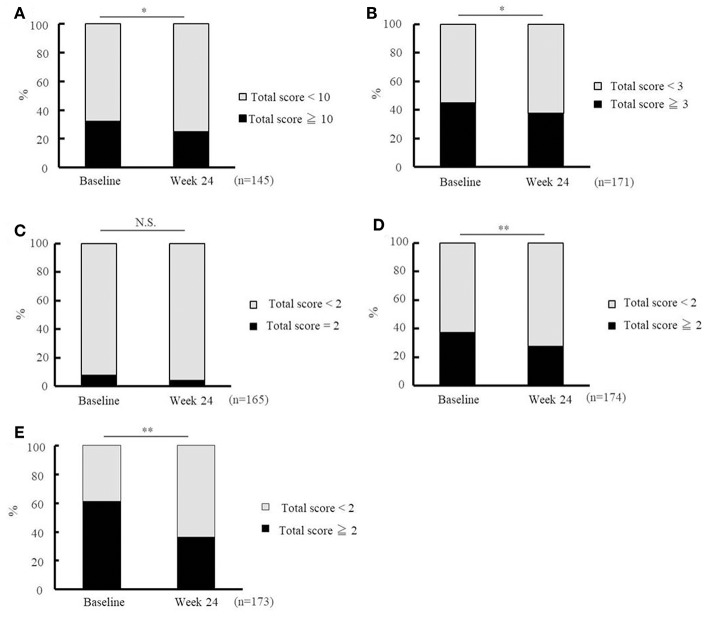
Effectiveness of Ninjin'yoeito as determined by Basic Checklist assessment. Changes in the risk ratio of care dependency accessed using the Basic Checklist after Ninjin'yoeito (NYT) administration: questions 1–20 for activities of daily living assessment **(A)**, questions 6–10 for motor function assessment **(B)**, questions 11 and 12 for nutritional assessment **(C)**, questions 13–15 for oral function assessment **(D)**, and questions 21–25 for depression assessment **(E)**. Data are presented as the mean ± standard deviation, ^*^p < 0.05 and ^**^p < 0.01 compared with the baseline as determined using the McNemar test. According to the criteria, a person with a total score of ≥ 10 for questions 1–20 (activities of daily living), ≥ 3 for questions 6–10 (motor function), = 2 for questions 11 and 12 (nutrition), ≥2 for questions 13–15 (oral function), or ≥2 for questions 21–25 (depression) was considered to be in need of care dependency; Basic Checklist, the 25-item Basic Checklist; (n), number of patients.

Figure 4 shows the percentages of patients who met the cut-off criteria for the five domains (activities of daily living, motor function, nutrition, oral function, and depression) at baseline and week 24. The percentage of patients who were considered to be in need of care dependency decreased significantly at week 24 in terms of activities of daily living (questions 1–20, Figure 4A), motor function (questions 6–10, Figure 4B), oral function (questions 13–15, Figure 4D), and depression (questions 21–25, Figure 4E). The changes observed in the nutrition domain (questions 11 and 12, Figure 4C) were not significant.

#### Overall Improvement

**Figure 5:**
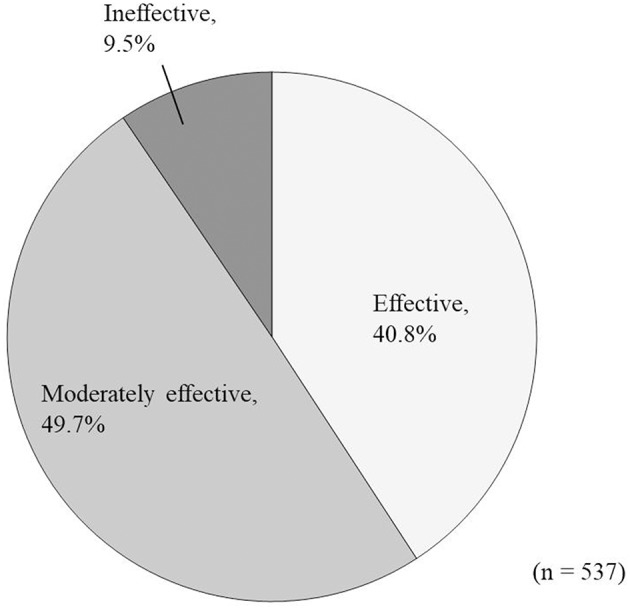
Overall improvement in response to Ninjin'yoeito administration according to physician assessment.

In the effectiveness analysis (*n* = 537), treatment was rated as “effective” in 219 patients (40.8%), “moderately effective” in 267 patients (49.7%), and “ineffective” in 51 (9.5%) patients (Figure 5).

## Discussion

At present there is no post-marketing survey available for NYT, and the present study is the first post-marketing reports for NYT. We found that the incidence of ADRs was 3.1% (Table 5A) and the major adverse reactions were gastrointestinal disorders (2.1%; Table 5B). Approximately 70% of ADRs in the cumulative incidence of adverse reactions occurred within 2 months after the administration of NYT.

VAS scores indicated that fatigue/malaise and anorexia scores improved after administration of NYT (Figure 2). According to the judgments of the physicians in charge, the severity scores were significantly improved in each of the following items: the deteriorated constitution after disease, night sweat, coldness of limbs, and anemia (Figure 3). Furthermore, we found that NYT increased BMI at week 24 especially in female patients. Interestingly, previous study suggested that mortality was related with the BMI score in older women with frailty (7). A recent report has indicated that NYT strengthened grip power in a comparative open-label trial in patients aged 65 and older (3). Moreover, anorexia and apathy improved following NYT administration in patients with dementia (8). These findings and those of the present trial indicate the beneficial effects of NYT in elderly people.

The Basic Checklist, a screening tool used to detect subjects in need of nursing care dependency, has many frailty-related aspects (9), as frailty is known to be closely related to the need of future care dependency. The satisfactory validity of the Basic Checklist has previously been verified by Kamegaya et al. (10), who followed 21,325 functionally independent elderly people for 3 years, and found that the subjects with more negative answers to Basic Checklist questions, particularly questions 1–20, had higher odds ratios for care dependency. In the present study, NYT improved the index of 1–20 in the Basic Checklist (Figure 4). These results indicate that NYT might have the potential to decrease the need for care dependency.

Questions 21–25 of the Basic Checklist are used for the detection of depression. In the present study, ratings were improved for all of these five questions, suggesting that NYT might reduce the risk of psychiatric/psychological frailty. According to a previous study (1), the depression ratings of Alzheimer's patients receiving both donepezil and NYT treatment, were significantly improved after 2 years treatment, by comparing with those receiving donepezil alone. In this context, the results of Kudoh et al. support our current findings.

As shown in this trial, NYT improved many Basic Checklist items, and it is thus possible that NYT not only prevents physical frailty but also psychiatric/psychological frailty.

Our study has some limitations, the most important of which is that there was no control group. Accordingly, further trials on the safety and efficacy of NYT are highly desirable.

## Conclusions

The findings of the present post-marketing survey for NYT indicate that the administration of this drug does not cause serious ADRs and might improve not only physical but also psychiatric/psychological frailty in elderly people. Further trials that include control groups are warranted.

## Author Contributions

All authors assisted in designing the study. SS wrote the initial draft of the manuscript. KS contributed to data analysis and interpretation, and assisted in the preparation of the manuscript. FA and MS contributed to data collection and interpretation, and critically reviewed the manuscript. All authors approved the final version of the manuscript, and agree to be accountable for all aspects of the work.

### Conflict of Interest Statement

The authors declare that the research was conducted in the absence of any commercial or financial relationships that could be construed as a potential conflict of interest.
